# Epidemiology and Individual, Household and Geographical Risk Factors of Podoconiosis in Ethiopia: Results from the First Nationwide Mapping

**DOI:** 10.4269/ajtmh.14-0446

**Published:** 2015-01-07

**Authors:** Kebede Deribe, Simon J. Brooker, Rachel L. Pullan, Heven Sime, Abeba Gebretsadik, Ashenafi Assefa, Amha Kebede, Asrat Hailu, Maria P. Rebollo, Oumer Shafi, Moses J. Bockarie, Abraham Aseffa, Richard Reithinger, Jorge Cano, Fikre Enquselassie, Melanie J. Newport, Gail Davey

**Affiliations:** Brighton and Sussex Medical School, Falmer, Brighton, United Kingdom; School of Public Health, Addis Ababa University, Addis Ababa, Ethiopia; Faculty of Infectious and Tropical Diseases, London School of Hygiene & Tropical Medicine, London, United Kingdom; Ethiopian Public Health Institute, Addis Ababa, Ethiopia; School of Medicine, Addis Ababa University, Addis Ababa, Ethiopia; Center for Neglected Tropical Diseases, Liverpool School of Tropical Medicine, Liverpool, United Kingdom; Federal Ministry of Health, Addis Ababa, Ethiopia; Armauer Hansen Research Institute/ALERT, Addis Ababa, Ethiopia; RTI International, Washington, DC

## Abstract

Although podoconiosis is one of the major causes of tropical lymphoedema and is endemic in Ethiopia its epidemiology and risk factors are poorly understood. Individual-level data for 129,959 individuals from 1,315 communities in 659 *woreda* (districts) were collected for a nationwide integrated survey of lymphatic filariasis and podoconiosis. Blood samples were tested for circulating *Wuchereria bancrofti* antigen using immunochromatographic card tests. A clinical algorithm was used to reach a diagnosis of podoconiosis by excluding other potential causes of lymphoedema of the lower limb. Bayesian multilevel models were used to identify individual and environmental risk factors. Overall, 8,110 of 129,959 (6.2%, 95% confidence interval [CI] 6.1–6.4%) surveyed individuals were identified with lymphoedema of the lower limb, of whom 5,253 (4.0%, 95% CI 3.9–4.1%) were confirmed to be podoconiosis cases. In multivariable analysis, being female, older, unmarried, washing the feet less frequently than daily, and being semiskilled or unemployed were significantly associated with increased risk of podoconiosis. Attending formal education and living in a house with a covered floor were associated with decreased risk of podoconiosis. Podoconiosis exhibits marked geographical variation across Ethiopia, with variation in risk associated with variation in rainfall, enhanced vegetation index, and altitude.

## Introduction

Podoconiosis (endemic non-filarial elephantiasis) is a non-infectious disease arising in barefoot individuals who are in long-term contact with irritant red clay soil of volcanic origin.^1^,^2^ The disease causes progressive bilateral swelling of the lowerlegs.^2^,^3^ Although the etiology is not fully understood, current evidence suggests that both genetic susceptibility and mineral particles from irritant volcanic soils play a role.^2^,^4^,^5^ Podoconiosis is the second most common cause of tropical lymphoedema after lymphatic filariasis (LF).^6^ Globally, it is estimated that there are 4 million people with podoconiosis, mainly in tropical Africa, Central and South America, and Southeast Asia.^3^,^4^ Previous studies have documented the adverse consequences of the disease,^7^–^11^ including reduced productivity.^7^ Other studies have documented that on average, most patients have five or more episodes of recurrent inflammatory swelling of their lymphedematous legs (“acute attacks”) and hence lose productive days.^12^–^14^ Severe stigma and discrimination are experienced by patients.^8^–^10^,^15^ The country with the presumed greatest burden of podoconiosis is Ethiopia, with an estimated one million people living with the disease.^4^

Simple lymphoedema management with foot hygiene using soap and antiseptic, bandaging, elevation of swollen legs during the night, and wearing protective shoes consistently make up the strategy to control podoconiosis.^3^,^16^ The first comprehensive epidemiological study of podoconiosis dates from 1969 and included 6,770 patients.^17^ Subsequently, Price conducted a range of epidemiological studies in east African countries and Cameroon.^1^ These studies excluded causes such as LF, leprosy, and onchocerciasis as a cause of podoconiosis.^17^–^21^ Subsequent studies documented the clinical features and pathology of the disease, a possible genetic susceptibility, and the role of soil particles and environment.^1^ After his extensive multidisciplinary work Price named the disease podoconiosis.^1^ In recent years, studies have documented the presence of podoconiosis in Ethiopia,^12^–^14^,^22^,^23^ Uganda,^24^ and Cameroon,^25^ and one has identified individual correlates.^26^ A study conducted in Ethiopia identified several individual correlates such as female sex, lower education, being unmarried, and having lower income.^26^ Price documented a variety of environmental correlates of podoconiosis,^1^,^27^ some of which have been confirmed through recent study of the environmental factors associated with podoconiosis, including the soil type, precipitation, and altitude.^28^ A recent study indicated that particles such as smectite, mica, and quartz within the soil were associated with podoconiosis prevalence.^28^

Although of great interest, each of these studies had limitations. Some studies relied on clinical diagnosis of podoconiosis without clear case definition. Important information about how possible differential diagnoses were excluded is missing in most of the studies. In earlier studies, the use of a purely descriptive approach without measuring important associations and confounding is another limitation. Although some studies identified individual or environment correlates no single study has simultaneously evaluated individual, household, climatic, and environmental variables to understand their relative contributions, or accounted for underlying spatial heterogeneity in disease occurrence.

We used data from the first national integrated LF and podoconiosis mapping survey conducted in Ethiopia in 2013 to describe podoconiosis epidemiology and determine individual, household, and environmental risk factors of the disease.

## Methods and materials

### Study setting.

Ethiopia is located in the Horn of Africa and has a surface area of some 1.1 million km^2^.^29^ According to the 2007 national population census, the total population was estimated at 73.8 million in 2007 and was projected to be 86.6 million in 2013.^29^,^30^ Ethiopia has great geographical diversity, with its topographic features ranging from 110 m below to 4,550 m above sea level.^30^ Ethiopia has a federal system of administration with nine regional states and two city administration councils.^31^ The regional states/city administrations are subdivided into zones, *woreda* (districts), *kebele* (sub-districts or municipalities), and *gotte* (villages). A *woreda* is the country's basic decentralized administrative unit and has an administrative council composed of elected members. The 817 *woreda* are further divided into about 16,253 *kebele*.

### Sampling strategy and participant selection.

This was a cross-sectional community-based study using convenience cluster sampling, conducted in seven regional states (Tigray, Affar, Amhara, Oromia, Somali, Southern Nations Nationalities, and Peoples [SNNP], and Harari) and two city administrations (Addis Ababa and Dire Dawa Administration Councils). Surveys for podoconiosis and LF were implemented simultaneously.

Two-stage cluster convenience sampling was used. The first stage identified high-risk *kebele* and then villages in each *kebele* were also purposively sampled, and the second stage sampled individuals within the chosen village. The primary sampling unit for the survey was the *kebele*. Two *kebele* were selected from each *woreda* based on reported history of lymphoedema cases collected through interviewing the *woreda* health officials, health providers, and village leaders 1 day before the survey. The secondary sampling unit was individuals selected from each village using systematic sampling from a random start point. Mobilization was conducted 1 day before the survey using health extension workers (community health workers of whom two on average are attached to each *kebele*). Every adult in the community was informed through a house-to-house visit that a survey would be conducted and was invited to participate.

On the day of the survey, all persons 15 years of age and above living in the selected communities were invited to participate in the study and were asked to gather at a convenient point, although details of the type of study were not provided to the community before meeting. The study objectives and procedures were then explained in the local language, and those willing to participate were asked to form two lines, one of men and the other of women. Based on the World Health Organization (WHO) mapping guideline for LF mapping^32^ 50 individuals were selected from each line using systematic sampling from a random start point, resulting in an overall sample of 50 males and 50 females. Two hundred individuals were therefore tested in each *woreda*. In most villages, it was possible to mobilize all adults in the community and obtain appropriate samples. Individuals were excluded from the study if they had not lived in the *woreda* for at least 10 years, had left the *woreda* for more than 6 months in the year before the survey, or did not provide informed consent.

### Data collection.

The data were collected between June and October 2013. Training of all survey coordinators, supervisors, and investigators in survey tools and procedures occurred over a 1-week period in June 2013. One day before the survey in each selected *kebele*, meetings were held with *kebele* leaders to explain the nature and purpose of the survey.

A podoconiosis questionnaire was adapted and expanded from previous studies conducted in Ethiopia^12^,^22^ and collected information on education, occupation, place of residence, shoe wearing practice, and distance to the nearest water source. Data were collected using android data capture by trained health workers. For individuals with lymphoedema, a checklist was used to identify possible differential diagnoses of podoconiosis. In this study, a confirmed podoconiosis case was defined as a person residing in the study *woreda* for at least 10 years, with lymphoedema of the lower limb present for more than 3 months for which other causes (LF, onchocerciasis, leprosy, Milroy syndrome, heart or liver failure) had been excluded. In those clinically confirmed to have podoconiosis, duration of illness, family history of similar illness among relatives, and disease stage according to the validated podoconiosis staging system^33^ were recorded.

Participants were requested to provide a finger-prick blood sample to be tested for circulating *Wuchereria bancrofti* antigen using an immunochromatographic card test (ICT) (BinaxNOW Filariasis, Inverness Medical, Scarborough, ME). The ICT kits were refrigerated while in storage in Addis Ababa, and central points in Hawassa, Dessie, and Mekele according to guidelines, and were kept in cool boxes throughout the fieldwork. An ICT was performed on all participants, and results were recorded with the individual's ID number both on the card, and on the Smartphone proforma. In villages where there was at least one positive ICT, all people with lymphoedema but with a negative ICT result were asked to provide 5 mL blood for antifilarial antibody (Wb123 assay) testing in Addis Ababa.

### Selection of environmental and climatic data.

**Figure 1.**
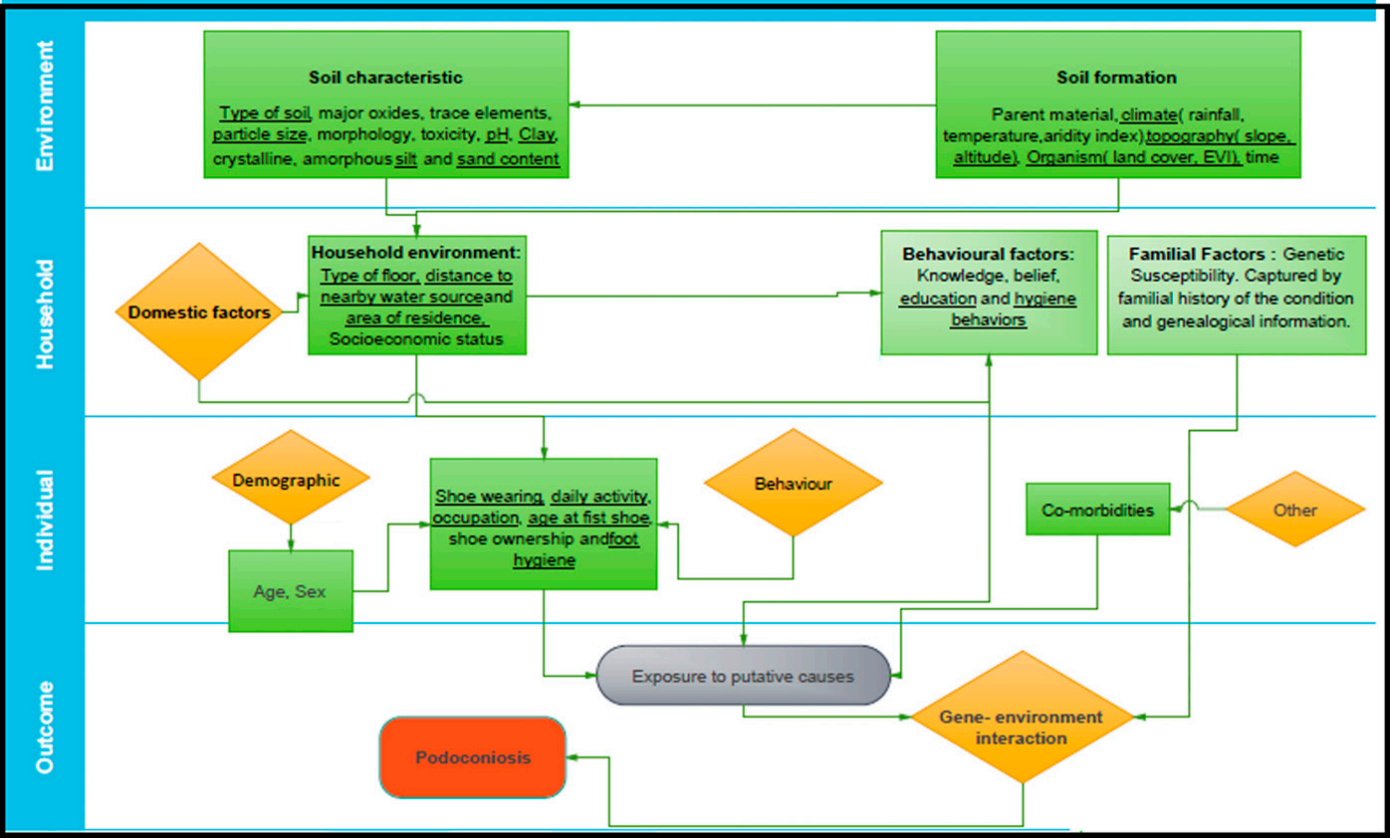
Conceptual framework to represent the relationship between environmental, household, and individual-level variables. Environmental variables such as climate, topography, flora, and fauna of the areas are important factors for generation of soil and to determine the characteristics of the soil. Soil physical and chemical properties including type of soil, particle size, morphology, pH, etc., are important factors, which either facilitate the penetration of skin barriers by putative mineral particles or increase exposure to them. Socioeconomic status affects household factors such as floor condition, access to water, and individual factors such as shoe ownership and foot hygiene practices, which in turn affect the risk of podoconiosis. Genetic susceptibility is an important factor in determining the outcome of the exposure, and is best measured by pedigree study. In the current study, we only measured the familial history of a similar condition. The underlined variables are those measured in the current analysis. The framework guided the analysis; principal component analysis was used to identify important covariates, which explained most of the variation among many multicollinear environmental and climate variables. Subsequently, a multilevel mixed-effects logistic regression model was developed using a likelihood-based approach, with random intercepts for village and *woreda*, for environment and individual variables separately, to identify candidate variables for the geostatistical model. Bayesian hierarchical models were developed to identify and measure the relative contributions of the risk factors for podoconiosis.

Environment and climate variables were selected based on a conceptual framework (Figure 1). First, variables that play an important role in weathering of rock and soil formation were selected, and then characteristics of soil thought to play an important role in the disease progression were selected based on the literature.^1^,^27^ Podoconiosis is caused by long-term barefoot exposure to red clay soil of volcanic origin^1^; thus, understanding how soil is formed is an important entry point in linking podoconiosis occurrence, the environment, and climate. There are five classic factors for soil formation: climate, topography, parent material, time, and organisms (flora and fauna).^34^–^36^ In addition, soil characteristics that facilitate exposure such as soil type, content, and particle size were considered as detailed in Supplemental Appendix File 1.

### Sources of satellite driven data.

The elevation data at 90 m^2^ resolution were extracted from a digital elevation model provided by the shuttle radar topography mission^37^ and these data were processed to calculate slope in degrees. The mean, minimum, and maximum atmospheric temperature, temperature in coldest and warmest quarters, and precipitation at 30-arcsecond (1 km) resolution were downloaded from the WorldClim website for the period 1950–2000 at 1 km^2^ spatial resolution.^38^ In addition, average and variance estimates of long-term land surface temperature (LST) at 1 km^2^ resolution were extracted from the African Soil Information System project.^39^

Euclidean distance to water bodies was calculated using SRTM Water Bodies data files at 250 m^2^ resolution.^40^ Land cover type was extracted from the qualitative global land cover map, defined within the United Nations (UN) land cover classification system using the environmental satellite (ENVISAT) mission's Medium Resolution Imaging Spectrometer (MERIS) sensor at 300 m^2^ resolution. Normalized difference vegetation index (NDVI) and enhanced vegetation index (EVI) at 250 m^2^ resolution were extracted from the African Soil Information System project.^39^ Population density was extracted from the AfriPop project at 90 m,^2^,^41^ and rural-urban classification at 1 km^2^ from the Global Rural-Urban Mapping project (GRUMP).^42^ Aridity index and potential evapotranspiration (PET) data were extracted from the Global-PET and Global-Aridity data sets (CGIARCSI), at 1 km^2^ resolution.^43^ Soil data including silt, clay and sand content, dominant soil type, and pH of the water at 1 km^2^ resolution were extracted from ISCRI and the African Soil Information System project.^44^ Finally, soil texture was extracted from the harmonized soil map of the world at 1 km^2^ resolution from FAO Geonetwork.^45^ Environmental data were extracted to cluster locations using ArcMap 10.0 (Environmental Systems Research Institute Inc. [ESRI], Redlands, CA).

### Data analysis.

Data were checked for accuracy daily and feedback was given to the field team the following day. After completion of the data collection, the data were downloaded in Excel (Microsoft Corp., Redmond, WA) format and imported to STATA 13.0 (Stata Corporation, College Station, TX) and further cleaned. Maps of the prevalence of podoconiosis were developed using ArcGIS 10.0 (ESRI).

Most of the climate and environmental variables showed multicollinearity. To deal with these challenges, we used exploratory principal components analysis that reduces correlated variables to a smaller set of uncorrelated variables, which explain most of the variation.^46^,^47^ The majority (92.7%) of the total variation was explained by the first two principal components, which had eigenvalues > 1 and were visually below the elbow of the scree plot. The first principal component explained 78.4% of the variance and two contrasting groups of variables emerged. Most of the variables had similar loading. The first group was related to temperature including LST, annual mean, minimum and maximum temperature, mean temperature in the coldest and warmest quarters, and annual PET. Altitude, aridity index, and rainfall were the contrasting variables. Mean annual temperature was selected from the first group based on the loading and literature.^48^ From the contrasting group, altitude was selected because other studies have repeatedly found it to be associated with podoconiosis.^28^,^48^ Annual precipitation, aridity index, and EVI showed the highest loads in the second principal component, which explained 13.6% of the variation. From the second component, EVI and annual precipitation were selected because both of them had equally high loading.

Selection of candidate variables for the geostatistical model was conducted using a multivariate multilevel mixed-effects logistic regression model using a likelihood-based approach, with random intercepts for *kebele* and *woreda*. All identified variables were initially included and non-significant (*P* > 0.1) variables sequentially removed using backward stepwise elimination to derive a minimally adequate model. Similarly, a model was developed to identify environmental factors associated with podoconiosis. Bayesian spatial geostatistical models were run in WinBUGS version 1.4.3 (MRC Biostatistics Unit, Cambridge and Imperial College London, UK), incorporating a geostatistical random effect. A burn-in of 10,000 iterations was allowed, followed by 20,000 iterations where values for monitored variables were stored and thinned by 10. Diagnostic tests for convergence of the monitored variables were undertaken, including visual examination of history and density plots. The runs were also assessed for evidence of autocorrelation. Model performance was assessed by comparing deviance information criteria; the details are presented in Supplemental Appendix File 1.

### Ethical considerations.

Ethical approval for the study was obtained from the Institutional Review Board of the Medical Faculty, Addis Ababa University, the Research Governance and Ethics Committee of Brighton and Sussex Medical School (BSMS), and ethics committees at the Ethiopian Health and Nutrition Research Institute (EHNRI) and Liverpool School of Tropical Medicine. Individual written informed consent was obtained from each participant ≥ 18 years of age. For those individuals < 18 years of age, consent was obtained from their parents/guardian and the participant themselves provided informed assent. Confirmed *W. bancrofti* infection was treated using albendazole (400 mg) and ivermectin (200 μg/kg body weight or as indicated by a dose-pole) according to WHO recommendations. For those with lymphoedema, health education was given about morbidity management.

## Results

**Figure 2.**
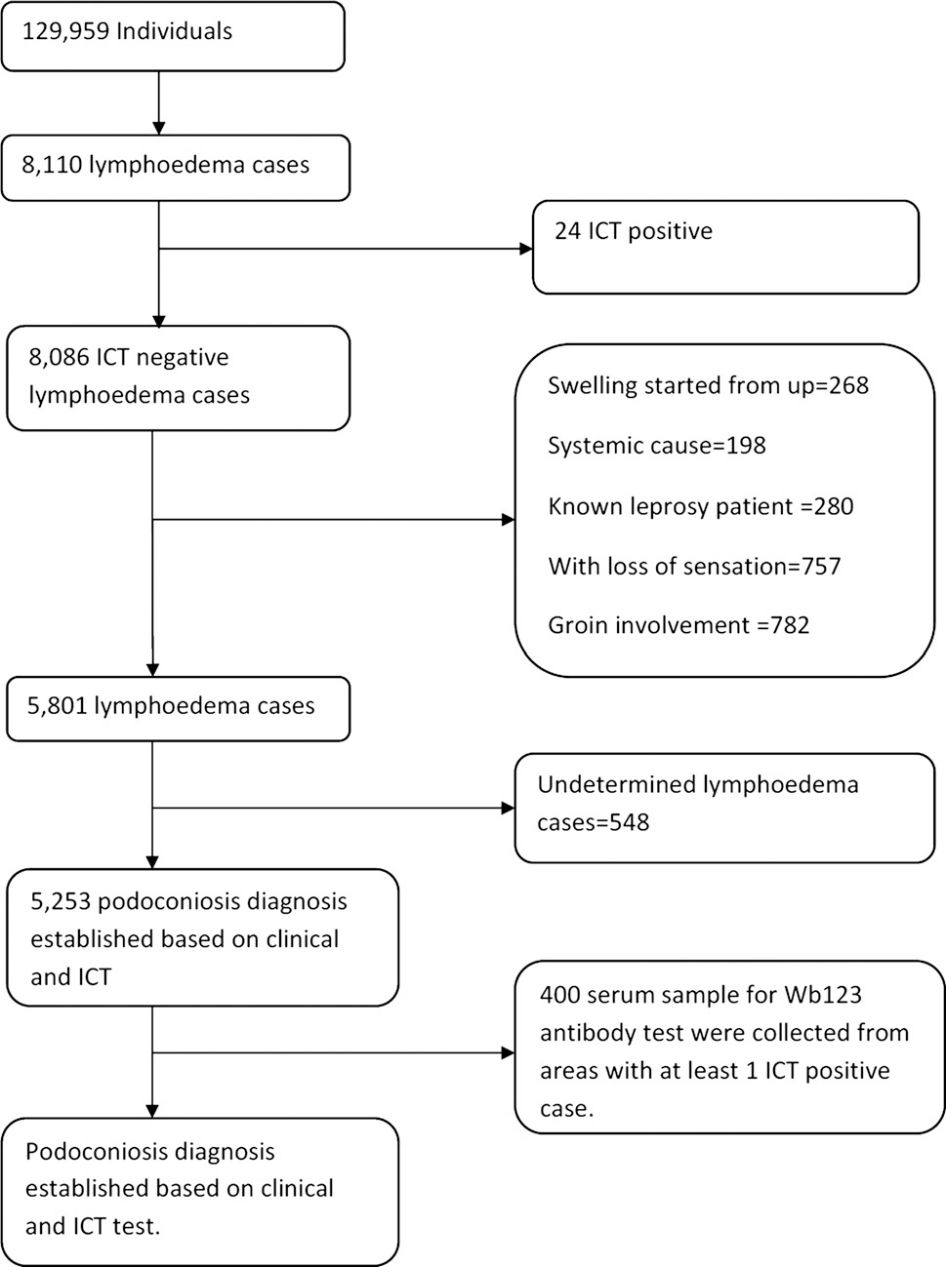
Podoconiosis diagnosis algorithm used in the national survey in Ethiopia 2013.

The survey was planned to be conducted in 692 (84.7%) of the 817 *woreda* of Ethiopia, and mapping was achieved in 659 (95.2%) of these. Thirty-three (4.8%) *woreda* were excluded or were inaccessible during the survey: 6 in Addis Ababa were excluded because of an exclusively urban population and almost universal shoe wearing practice, 23 in Somali were inaccessible as a result of conflict, and 1 in Oromia and 3 in Amhara had been merged or double counted. Individual-level data were available for 129,959 individuals from 1,315 villages in 659 *woreda* (Figure 2). The median age of the participants was 34 years (interquartile range [IQR], 25 to 46). The male/female ratio of the respondents was 1.0:1.0, reflecting the selection process. The mean number of individuals per *kebele* was 98.

**Figure 3.**
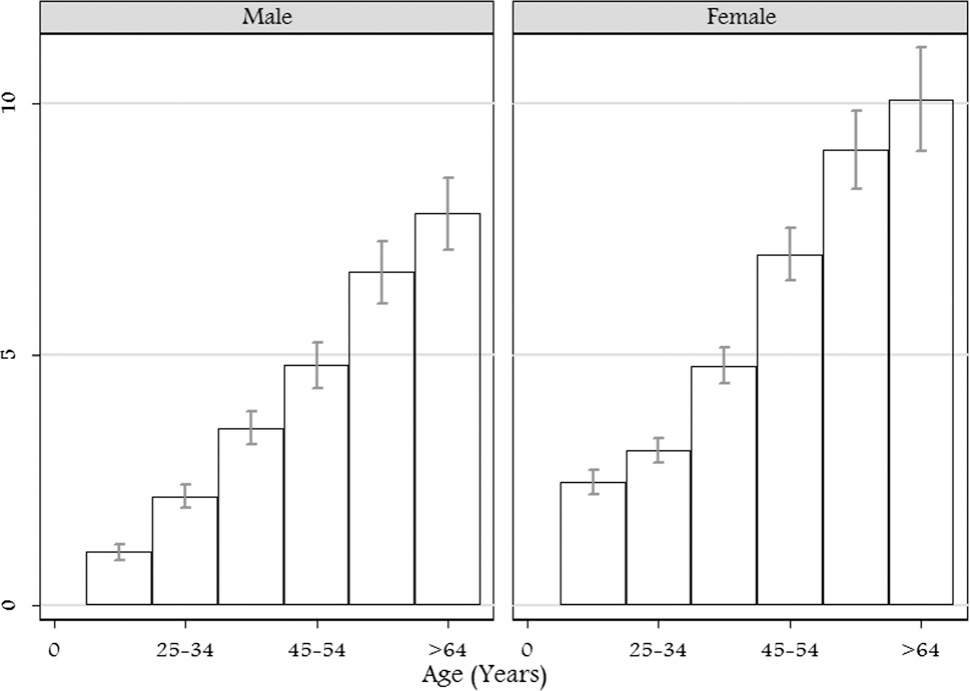
Age and sex disaggregated prevalence of podoconiosis among adults 15 years of age and above in Ethiopia. The two graphs show that as age increases, the prevalence of podoconiosis increases. Higher prevalence is recorded among females in all age categories as compared to males.

In total, 8,110 individuals with lymphoedema of the lower limb were identified. The overall prevalence of lower limb lymphoedema was 6.2% (95% confidence interval [CI] 6.1–6.4%), with a significant difference in prevalence between men (5.3%, 95% CI 5.2–5.5%), and women (7.1%, 95% CI 6.9–7.3%). Of the 8,110 lymphoedema cases of the lower limb, 24 (0.3%) were found to be ICT positive, 268 (3.3%) had swelling of the descending type, starting from higher up in the leg, and 782 (9.6%) had groin involvement. Based on the clinical algorithm for podoconiosis diagnosis a total of 2,833 lymphoedema cases were considered because of other etiologies and were consequently excluded from the analyses. Thus, the total number of people with podoconiosis was 5,253 (Figure 2), with overall podoconiosis prevalence being 4.0% (95% CI; 3.9–4.1%); prevalence among men and women was 3.4% (95% CI 3.3–3.5%), and 4.7% (95% CI 4.5–4.8%), respectively, and prevalence increased with age (*P* < 0.001) (Figure 3).

### Characteristics of people with podoconiosis.

Of the 5,253 people with podoconiosis, the male/female ratio was 0.7:1. The majority of affected people were in the age group 35–54 years, whereas only 10.2% were in the age group 15–24 years. The mean age at first noticing the swelling was 25.0 (± SD 14.6; range: 5–87) years. On average, women noticed swelling earlier (23.9, SD ± 13.8) than men (26.6, SD ± 15.5, *P* < 0.001). Only 12.0% had noticed swelling when younger than 10 years of age, whereas 8.1% first noticed the swelling when older than 50 years of age. More than half (52.0%) noticed leg swelling while they were between the ages of 10 and 30 years (Table 1). On average, people with podoconiosis had lived with leg swelling for 19.8 (SD ± 14.2) years, men for 20.9 (SD ± 14.62) years, and women for 19.1 (SD ± 13.9) years (*P* < 0.001) and 22.7% had or remembered at least one blood relative with a similar condition. The majority (48.8%) of people with podoconiosis had stage two disease (Table 1); there was no significant difference in the distribution of disease stage among men and women.

### Podoconiosis-preventive behaviors.

Among all respondents, most (89.6%) had at some time worn shoes and 85.2% of them were wearing shoes during the interview. Regarding the type of shoe, most (50.7%) were wearing hard plastic shoes, followed by open sandals (32.0%), leather shoes (12.8%), canvas shoes (4.1%), and other types of shoe (0.39%). Only 57.9% of the respondents were wearing protective (enclosed) shoes during the interview. The mean age at first shoe wearing was 11.8 years (SD 9.6) with a slight difference between men (mean ± SD, 12.1 ± 9.8) and women (11.4 ± 9.4 women, *P* < 0.001). On average, the participants had worn shoes for 24.1 (SD; 13.6) years. More men (87.0, 95% CI; 86.8–87.3%) than women (83.3, 95% CI; 83.0 to 83.5) were wearing shoes at the time of the interview. There was no significant difference in protective shoe wearing between men (58.3, 95% CI; 57.9–58.7) and women (57.6, 95% CI; 57.2–57.9). Most respondents wore shoes when going to market (98.2%) or when walking far (97.2%), and fewer, though still substantial numbers, during the rainy season (90.6%), at home (89.8%), and when going to the field (80.1%). Most (69.5%) of the participants practiced foot hygiene (washing the feet with soap) on a daily basis, whereas 25.4% practiced foot hygiene twice per week.

### Geographical distribution of podoconiosis.

**Figure 4.**
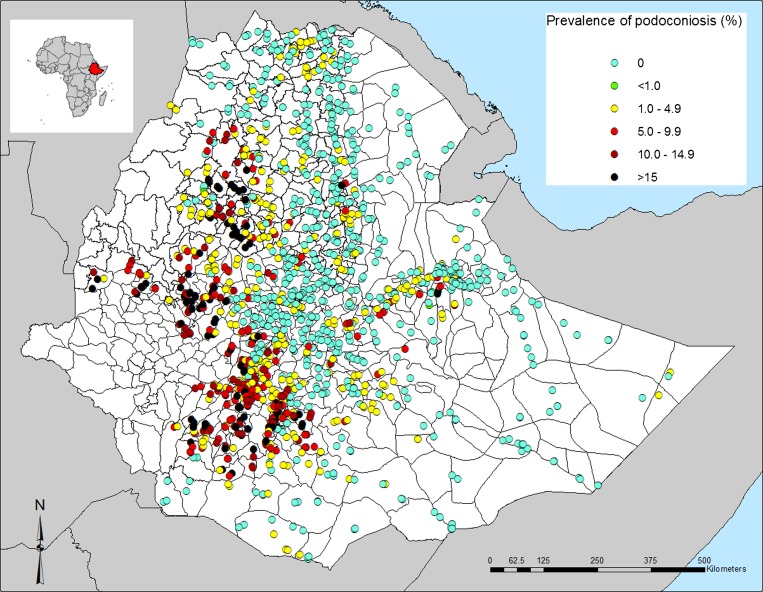
The geographical distribution of the prevalence of podoconiosis among adults ≥ 15 years of age in Ethiopia, 2013.

Figure 4 presents the prevalence of podoconiosis by survey cluster and shows marked geographical variation, with prevalence ranging from 0% to 54.6%. Clusters of high prevalence (> 5%, denoted in red and black in Figure 4) were exclusively found in Amhara, Oromia, and SNNP regional states, which represent most of the central highlands of Ethiopia. Most of the eastern and far northern part of the country had zero or near zero prevalence. All of the clusters in Addis Ababa, Affar, Dire Dawa, and Harari had zero prevalence of podoconiosis. Few cases were identified in Tigray and Somali regional states. The southwestern part of the country also had high prevalence clusters.

### Risk factors.

Summary of the environmental factors is presented in (Table 2). In the final non-spatial model, both individual and environmental factors were found to be risk factors for podoconiosis. Thus, individual-level factors associated with an increased risk for podoconiosis included female gender (odds ratio [OR] = 1.3; 95% Bayesian credible interval [BCI]; 1.2–1.4), age (OR = 1.02; 95% BCI; 1.02–1.03), unmarried status (OR = 1.4; 95% BCI; 1.3–1.5), religion; factors associated with a decreased risk included secondary or higher education, increased foot hygiene, employment, housing with covered floor (OR = 0.3; 95% BCI; 0.3–0.4). Among individual factors there was no significant association between shoe wearing and risk of podoconiosis. Three environmental variables were found to be associated with risk for podoconiosis after accounting for spatial dependence: rainfall, EVI, and altitude (Table 3). 

The geostatistical models also indicated that podoconiosis risk can be moderately variable over relatively short distances, with spatial variances of 0.25 and an estimated spatial range of roughly 31 km.

## Discussion

Understanding the epidemiology of a disease is the first step in designing control and prevention strategies. For podoconiosis, such data have to date been derived from small-scale studies. We present here the epidemiology of podoconiosis using nationwide data from Ethiopia. These were gathered using a predefined clinical algorithm and a panel of diagnostic approaches to exclude other potential causes of lower limb lymphoedema. In addition, a robust modeling approach was used to identify individual and environmental risk factors of podoconiosis among adults 15 years of age and above. Podoconiosis accounted for 64.8% of cases of lymphoedema of the lower limb. The prevalence of podoconiosis in surveyed high-risk communities was 4.0%, with higher prevalence among women and older age groups. A number of risk factors were identified in our analysis including sex, age, foot hygiene practice, education, and type of floor. In addition, environmental and climate variables (rainfall, altitude, and EVI) determined the large scale risk of podoconiosis across Ethiopia.

At 4.0%, the prevalence in high-risk communities observed in our study is higher than previous national level podoconiosis estimates. Our recent review of published work estimated a national prevalence of 3.4%,^49^ with older pioneering studies suggesting a prevalence of 2.7% (by Ooman in 1969^17^) and 2.8% (by Price in 1974^19^). Recent studies in Ethiopia and elsewhere show the high prevalence of podoconiosis in certain areas.^12^–^14^,^22^,^23^,^50^ Thus, five studies conducted in north, central, southern, and western Ethiopia between 2000 and 2012 reported prevalence ranging from 2.8% to 7.4%.^12^,^13^,^22^,^23^,^50^ A recent study conducted in Uganda in high-risk communities documented 4.5% prevalence,^24^ whereas another in Cameroon documented 8.1% prevalence.^25^ The difference between our estimate and previous estimates is largely attributed to our survey's sampling methodology and larger sample size.

Female sex was found to be associated with increased risk of podoconiosis. Other studies have yielded inconsistent findings surrounding the sex ratio in podoconiosis. The only other study that adjusted for covariates showed that women had increased odds of podoconiosis compared with men (OR = 3.01; 95% CI: 1.73 to 5.25).^26^ Two studies conducted in western Ethiopia documented male/female ratios of 0.7:1^14^ and 0.5:1,^12^ and another, in Cameroon, recorded a ratio of 0.5:1.^25^ Most of the other studies found an equal sex ratio—1:1 in southern^22^ and northern^23^ Ethiopia, and in Uganda.^24^ The only contemporary study that found men outnumbering women was conducted in central Ethiopia and indicated a sex ratio of 1.2:1.^13^ Our finding here is not unexpected, given sex differences in preventive behaviors such as shoe wearing practices between men and women. Equally, gender imbalances between men and women may influence access to resources including shoes and socks. In addition to these social factors that may mediate the effect of gender on disease risk, differences in genetic susceptibility^5^ and biological, particularly hormonal, influences may also be important.

**Figure 5.**
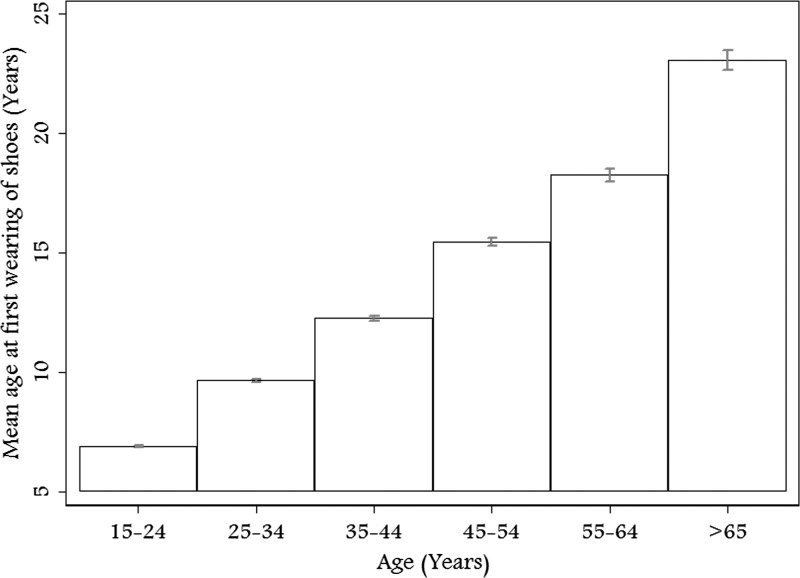
Graph showing age at first shoe use by age among adults 15 years of age and above in Ethiopia. The graph shows a decreasing secular trend of age at first wearing shoes: the younger age groups tend to start wearing shoes at an earlier age than the older age groups.

In our study, the prevalence of podoconiosis increased steadily with age, and high prevalence of podoconiosis was found among individuals 65 years of age or above. Previous studies in Ethiopia have also documented prevalence increasing with age,^12^,^14^,^20^,^22^,^23^,^26^ as have studies in Uganda^24^ and Cameroon.^25^ Given that podoconiosis is a chronic condition and that most patients and health professionals are unaware that treatment is possible, it is to be expected that prevalence increases with age. Other factors that may be important include cumulative exposure to the putative causes over an individual's life and changes in shoe wearing practices. This study indicates a linear decrease in the age at first shoe use by age category (Figure 5

), that is, there is a secular trend toward earlier shoe wearing in the younger age groups.

Foot hygiene practices were associated with podoconiosis disease status. These findings further strengthen the importance of promotion of these behaviors in preventing podoconiosis. The role of water, sanitation, and hygiene (WASH) in neglected tropical disease (NTD) control has been gaining much attention recently.^51^ Studies have indicated the interplay between NTDs and WASH,^51^ and this study adds to the evidence. Shoe wearing was not found to be associated with podoconiosis. The explanation for this may be reverse causality, in that people with podoconiosis tend to start wearing shoes after developing the disease either to prevent progression of disease or to conceal the swelling. Living in a house with an uncovered (mud or earth) floor was associated with podoconiosis, which may reflect either the importance of indoor exposure in addition to outdoor exposure or the influence of socioeconomic status of the individual. Occupation was also found to be associated with podoconiosis. Occupation may be related to the underlying socioeconomic status of individuals, which in turn may affect individual access to shoes, water, and foot hygiene practices or more broadly access to information that leads to preventive behaviors. In our study, people with podoconiosis were less likely to be married or to have attended formal schooling. The interplay between podoconiosis stigma and mate selection has been described in qualitative studies. As a result of a widespread misconception that podoconiosis runs within families whatever the environmental exposure, people with podoconiosis experience great difficulty in finding a marriage partner, particularly one without podoconiosis.^8^,^52^ The interaction between podoconiosis and education may be bidirectional. People with podoconiosis may have less access to education because of a lack of finances, (itself caused by reduced productivity^7^), attendant illness such as acute attacks,^12^ or dropout from school as a consequence of stigma and discrimination.^8^,^12^,^52^ Equally, more educated people may be more informed of how to prevent podoconiosis and may have more access to shoes and water.

There are two prevention strategies for podoconiosis. The first is early and consistent protective shoe wearing.^2^,^4^ The prevalence of shoe wearing was 85.2%, which is considerably higher than that reported in northern Ethiopia in 2011 (76.4%)^23^ and in southern Ethiopia in 2007 (55.2%).^9^ However, only 60% of respondents were wearing protective shoes, and only 60% had worn shoes before 12 years of age. Although the age at first use of shoes is diminishing, the rate of protective shoe use needs more attention, as only half of shoe-wearers are using such shoes. Because our study was conducted during the rainy season, it is unlikely that the rate of open shoe wearing is related to weather conditions as reported elsewhere.^53^ An encouraging result is that age at first shoe wearing is significantly lower in the younger age groups (Figure 5). The second prevention strategy is washing the feet with soap and water. Although on average the respondents have reasonable access to water,^14^ of those who were barefoot during the interview, one-third (29.4%) were not washing their feet on a daily basis. Such behaviors may further expose barefooted individuals to podoconiosis.

After controlling for the previous individual factors, rainfall, altitude, and EVI were found to be associated with risk of podoconiosis. At this scale, these environmental factors may affect the risk of podoconiosis through influencing soil formation, or by affecting human exposure to the soil. Annual rainfall > 1,000 mm was associated with increased risk podoconiosis^48^,^49^ in previous studies. Rainfall may play an important role in podoconiosis occurrence; previous studies have indicated the potential association of rainfall and podoconiosis. First, rainfall is one of the factors that governs the generation of soil. Moisture is an important factor that facilitates weathering and leaching. Second, rainfall may play an important role in exposure to mineral particles linked to podoconiosis, by producing sticky mud, which increases the contact time with the soil. Previous studies have indicated that soils associated with podoconiosis are slippery and adhesive if allowed to dry. Such occlusive adhesion encourages absorption of the particles by increasing exposure time.^48^ The topography of the land affects weathering and soil formation. Altitude governs temperature, rainfall, and vegetation of an area, all of which play an important role in weathering and soil formation in an area. Previous studies have also indicated the potential association of podoconiosis and altitude, suggesting that podoconiosis was common in areas with altitude > 1,000 m above sea level.^54^ Our finding indicates that the greener, more vegetated areas had a lower risk of podoconiosis; this may be associated with decreased exposure to the mineral particles linked to podoconiosis in areas with high vegetation cover.

The current study has several strengths. First, it used a predefined clinical algorithm to exclude other causes of lower limb lymphoedema. Second, we used ICT and Wb123 diagnostic tests to exclude lymphoedema caused by lymphatic filariasis. Third, we were able to sample almost all previously unmapped *woreda* in Ethiopia, yielding a large sample size and extensive geographical coverage. Despite the robust approach used to analyze and model these data, a number of caveats have to be outlined. Currently, there is no confirmatory test for podoconiosis; hence, our diagnosis was based on excluding other etiologies and identifying clinical features specific to podoconiosis. Although this technique has been found to have excellent predictive value in endemic communities,^22^ it has not been validated in non-endemic communities. Our sampling approach included use of anecdotal reports of LF and podoconiosis at *kebele* level, which is likely to lead to an overestimate of the number of lymphedema cases. Furthermore, individual selection was done after gathering the village community centrally, potentially leading to self-selection bias, as individuals with lymphoedema may come forward preferentially. However, we achieved high turnout of individuals in these communities through house-to-house mobilization, minimizing selection bias. We note that research on filariasis treatment coverage estimates in Haiti found little difference between coverage estimates obtained through a convenience sample of houses near distribution points compared with a cluster survey.^55^

The prevention, control, and treatment of podoconiosis are important components of the health and development of endemic countries. The importance of NTD prevention and control as a development agenda has been documented.^56^ Previous studies have estimated the economic impact of podoconiosis, which reduces individuals' productivity by half,^7^ and its social consequences, including stigma and discrimination.^8^–^10^,^52^ Understanding the geographical distribution of podoconiosis is very important in identifying priority areas, targeting control, planning for elimination, and monitoring progress. Until now, such data have been lacking for podoconiosis, and where they do exist, are old, suffer diagnostic inaccuracies, and do not reflect the current status of the disease. Up-to-date, reliable information at high resolution is urgently required in Ethiopia and in other endemic countries to guide interventions.^57^ The next critical step will be to identify the environmental limits of podoconiosis, estimate the population at risk, and predict the prevalence using robust models such as Bayesian model-based geostatistical approaches^58^ to predict continuous distribution of podoconiosis while measuring the uncertainties and accounting for spatial dependency.

In conclusion, we report here the epidemiology of podoconiosis in Ethiopia. The results show a high burden of podoconiosis in Ethiopia influenced by both individual and environmental factors. Our findings will help in future risk mapping of podoconiosis using environmental variables, and provides important information for decision-makers to prioritize interventions to high-risk individuals. Our results will serve as a baseline for the Ethiopian Federal Ministry of Health in podoconiosis programming and resource allocation.

## Supplementary Material

Supplemental Datas.

## Figures and Tables

**Table 1:** Descriptive statistics of people with podoconiosis ≥ 15 years of age in Ethiopia*

Variable	Number (%)
Gender Female	3045 (58.0)
Male	2208 (42.0)
Age group: 15–24	538 (10.24)
25–34	918 (17.48)
35–44	1119 (21.30)
45–54	1064 (20.26)
55–64	868 (16.52)
> = 65	746 (14.20)
Marital status	
Unmarried	557 (10.6)
Married	3722 (70.9)
Divorced	272 (5.2)
Widowed	702 (13.4)
Duration of podoconiosis Mean (SD)	25.04 (14.58)
Shoe wearing duration in years Mean (SD)	22.42 (14.76)
Disease stage	
Stage 1	875 (16.7)
Stage 2	2562 (48.8)
Stage 3	1399 (26.6)
Stage 4	308 (5.9)
Stage 5	109 (2.1)
Occupation professional^1^	68 (1.3)
Semiskilled^2^	3488 (66.2)
Unemployed^3^	1705 (32.5)
Residence rural	4467 (85.0)
Urban	786 (15.0)
Ever attended school	
Yes	1087 (20.7)
No	4166 (79.3)
Literacy no formal education	4166 (79.3)
Primary (1–8)	906 (17.2)
Secondary (9+)	181 (3.4)
Family history of leg swelling	
Yes	1192 (22.7)
No	4061 (77.3)
Number in family with leg swelling, dead or alive (*N* = 1192), Mean (SD)	1.44 (1.73)
1	857 (71.9)
2	222 (18.6)
3	74 (6.2)
>3	39 (3.3)
Age at first noticing the swelling Mean (SD)	24.67 (15.06)
< 10	630 (12.0)
10–19	1395 (26.6)
20–29	1335 (25.4)
30–39	898 (17.1)
40–49	567 (10.8)
≥ 50	428 (8.1)
Years lived with swelling	
< 10	1817 (34.6)
10–19	1478 (28.1)
20–29	918 (17.5)
30–39	555 (10.6)
40–49	318 (6.1)
> = 50	167 (3.2)

* In total 5,253 individuals with podoconiosis were identified among 129,559 surveyed individuals.

1 Certified professional.

2 Not-certified.

3 Currently not employed and dependent on others for living.

**Table 2:** Summary statistics of the reduced set of climatic and environmental covariates included in model building

Variable	Median (range)*
Climate	
Mean annual temperature (°C)	19.0 (10.0–31.0)
Annual rainfall (mm)	1042 (139–2090)
Environmental	
Altitude (meters)	1895 (−105 to 3238)
Savannah or Grasslands† (%)	27.67%
Urban classification (%)	26.8% urban
Population density (km^2)^	129.0 (0.82–92863)
Enhanced vegetation index (EVI)	0.27 (0.04–0.56)
Distance to nearest surface water (km)	6.4 (0–144)
Fine soil texture (%)	43.13%
pH of the water in the soil	6.20 (4.60–9.3)
Slope of the land (°)	1.67 (0.01–17.75)
Clay content (%)	35.5 (17–60)
Sand content (%)	30 (10–71)
Silt content (%)	32 (9–50)
High activity soil (%)	76.56%

* Proportion of sites for binary variables (Savannah/Grasslands, urban classification, fine soil texture, clay content, sand content, silt content, heavy activity soil).

† Reclassified from global land cover.

**Table 3:** Adjusted odds m of individual, household, and geographical risk factors of podoconiosis using a Bayesian model and data from 1,313 villages throughout Ethiopia

Variable	Category	Adjusted 95% Bayesian credible intervals (BCI)
		OR (95% BCI)
Sex	Male	1.0
	Female	1.3 (1.2–1.4)*
Age in years (continuous)		1.02 (1.02–1.03)*
Education	No formal education	1.0
	Primary 1–8	0.6 (0.6–0.7)*
	Secondary 9–12	0.3 (0.3–0.4)*
	Post-secondary > 2	0.1 (0.1–0.2)*
Marital status	Married	1.0
	Unmarried	1.4 (1.3–1.5)*
Religion	Muslim	1.0
	Other	3.4 (3.1–3.6)*
How often do you wash your legs?	Daily or more often	1.0
	Two-three times a week	0.9 (0.8–1.0)
	Weekly or less often	2.9 (2.4–3.4)*
Occupation:	Professional	1.0
	Semiskilled	2.4 (1.9–3.0)*
	Unemployed	2.2 (1.7–2.8)*
Type of floor	Mud/earth	1.0
	Cement/wood/plastic	0.3 (0.3–0.4)*
Enhanced vegetation index (EVI)	< 0.2	1.0
	0.2–0.4	0.6 (0.5–0.6)*
	> 0.4	0.4 (0.3–0.4)*
Mean annual rainfall	1,000	1.0
	> = 1,000	1.1 (1.0–1.1)*
Altitude	< 1,000	1.0
	1,000–2,800	1.3 (1.2–1.4)*
	> 2,800	1.8 (1.5–2.1)*
Range of the spatial effect [range in km]		31.1 (6.1–198.0)
Spatial variance		0.253 (0.252–0.490)

* Significant.
