# Supplementary material for: Epidemiology and Individual, Household and Geographical Risk Factors of Podoconiosis in Ethiopia: Results from the First Nationwide Mapping
Source: Am J Trop Med Hyg. 2015 Jan 7;92(1):148–58. doi: 10.4269/ajtmh.14-0446 (PMC4288951; doi:10.4269/ajtmh.14-0446)
Supplement: Supplementary file 1 [file SD5.pdf]

SUPPLEMENTAL APPENDIX

EPIDEMIOLOGY AND INDIVIDUAL, HOUSEHOLD, AND GEOGRAPHICAL RISK FACTORS OF PODOCONIOSIS IN ETHIOPIA: RESULTS FROM THE FIRST NATIONWIDE MAPPING

BACKGROUND TO SELECTION OF ENVIRONMENTAL VARIABLES

Podoconiosis is caused by long-term barefoot exposure to red clay soil of volcanic origin[1]; thus, understanding how soil is formed is an important entry point in linking podoconiosis occurrence, the environment, and climate. There are five classic factors for soil formation: climate, topography, parent material, time, and organisms (flora and fauna).[2–4] Soil varies depending on a range of climatic conditions[5]: temperature and precipitation influence the degree of weathering and leaching.[3] Seasonal and daily variability of temperature affects chemical reactions, moisture, biological activity, and vegetation type, through influences on weathering.[6] The aridity of an area plays an important role, and winds in arid zones may redistribute sands and other soil particles.

Topography of the land, both altitude and slope, affects weathering. Altitude governs temperature, rainfall, and vegetation of an area. Areas with steep slopes facing the sun are warmer, in addition, soils on slopes may more easily be eroded than soils on level ground. Plants, animals, microorganisms, and human beings affect soil weathering.[3] Human activities such as erosion and deforestation have significant impact on soil formation.[7] Few soils weather directly from the underlying rock, and may therefore have completely different chemical and physical properties, making underlying rock a poor predictor of overlying soil type.[6] Fine particles have been linked to podoconiosis occurrence,[8,9] and are thought to easily penetrate the skin and enter the body. Other factors such as distance to surface water are likely to be relevant to preventive behaviors such as foot hygiene, and therefore were included, as was urban-rural variation.

MULTICOLLINEARITY

Most of the climate and environmental variables showed multicollinearity (Figure S1 and Supplemental Table 1). To deal with these challenges, we used exploratory principal components analysis (PCA). Exploratory PCA is used to replace correlated variables with a smaller number of uncorrelated variables, which explain most of the variation.[10] The PCA is often used as a pre-processing step for subsequent analyses.[11]

As a rule of thumb, it is recommended to select the first k components, which explain 70–80% of the variation,[10] and to retain components with eigenvalues > 0.7.[12] In the next step, the extracted factors were interpreted for their loadings, using the recommended cut-offs for factor loadings (coefficients) as follows: variables with loadings ≥ 0.60 were considered to be heavily loaded, those with loadings 0.40–0.59 to be moderately loaded, and those with coefficient 0.3–0.39 to be modestly loaded.[13] The selection of variables from each component was based on the PCA loading and the literature (Supplemental Table 2). Altitude was selected as a base variable because it appeared to be an important component of soil formation pathways and determines many of the climate variables.[14,15] In addition, it was consistently found to be associated with the occurrence of podoconiosis. The PCA analysis was conducted using 11 climate and environmental variables. The majority (92.7%) of the total variation was explained by the first two principal components, which had eigenvalues greater than one and were below the elbow of the scree plot (Figure S2). The first principal component explained 78.4% of the variance and two contrasting groups of variables emerged. Most of the variables had similar loading. The first group was related to temperature including land surface temperature, annual mean, minimum and maximum temperature, mean temperature in the coldest and warmest quarters, and annual potential evapotranspiration. Altitude, aridity index, and rainfall were the contrasting variables. Mean annual temperature was selected from the first group based on the loading and literature. From the contrasting group, altitude was selected because it was found to be associated with podoconiosis. Annual precipitation, aridity index, and enhanced vegetation index (EVI) showed the highest load in the second principal component, which explained 13.6% of the variation. From the first component, EVI and annual rainfall were selected because both of them had the highest loading.

BAYESIAN MODELS

Final equivalent Bayesian models were run in WinBUGS version 1.4 (MRC Biostatistics Unit, Cambridge and Imperial College London, UK), incorporating a geostatistical random effect. Models took the form:

$$Y_{ij} \sim Bernoulli(p_{ij}),$$

$$\text{logit}(p_{ij}) = \alpha + \sum_{i=1}^{p} \beta_i \times x_i + \sum_{j=1}^{p} \beta_j \times x_{i,j} + v_i + u_i,$$

where Y_{ij} is the disease status of individual i in cluster j, p_{ij} is the probability of a positive response, α is the intercept, $\sum_{j=1}^{p} \beta_i \times x_i$ is a vector of independent variables at the individual level measured in the field multiplied by their coefficient β_i, $\sum_{j=1}^{p} \beta_j \times x_{i,j}$ is a vector of independent variables at the cluster level multiplied by their coefficient β_j, v_i is a non-spatial random effect (NSRE) and u_i is a spatial random effect (SRE). Non-informative priors were specified for the intercept (uniform prior with bounds $-\infty,\infty$) and the coefficients (normal prior with mean = 0 and precision, the inverse of variance = 1×10^6). The NSREs were incorporated into all models, to allow residual variation to have uncorrelated and correlated components. The SRE models any residual correlation that is spatially structured using a stationary exponential decay function. The NSRE had a non-informative priors imposed on its variance (uniform distribution with delimiting values of 0.01 and 100).

A burn-in of 10,000 iterations was allowed, followed by 20,000 iterations where values for monitored variables were stored and thinned by 10. Diagnostic tests for convergence of the monitored variables were undertaken, including visual examination of history and density plots. The runs were also assessed for evidence of autocorrelation. Model performance was assessed by comparing deviance information criteria (Supplemental Table 3).

SEMIVARIOGRAM ANALYSES

To test for the presence of spatial autocorrelation of podoconiosis prevalence, semivariogram analysis was employed (Figure S3). This analysis estimates the spatial autocorrelation structure of a variable by defining semivariance (a measure of expected dissimilarity between a given pair of observations made at different locations in space) as a function of lag (the distance separating the observation locations). If spatial autocorrelation is evident, semivariance typically rises with distance; eventually plateauing to a maximum value termed the sill. The separation distance at which the sill is reached is termed the range and represents the maximum distance over which values are autocorrelated, with larger separation distances implying spatial independence. Details of semivariogram use are described very well elsewhere.[16,17] Empirical semivariograms were developed from the raw data. Semivariogram analyses were run from R version 3.0.2.

REFERENCES

1. Price E, 1990. *Podoconiosis: Non-Filarial Elephantiasis*. Oxford: Oxford Medical Publications.
2. Amirossadat Z, 2013. Genesis and classification of soils in Segzi Plain (Iran). *IJSRK 1:* 1–12.
3. Soil Survey Staff, 2006. *Keys to Soil Taxonomy*. Tenth edition. Washington, DC: U.S. Department of Agriculture, Natural Resources Conservation Service.
4. Jenny H, 1941. *Factors of Soil Formation*. New York: McGraw-Hill.
5. Liu F, Geng XY, Zhu A-X, Fraser W, Waddell A, 2012. Soil texture mapping over low relief areas using land surface feedback dynamic patterns extracted from MODIS. *Geoderma 171–172:* 44–45.
6. Social Classification Working Group, 1998. *The Canadian System of Soil Classification*. Third edition. Canada: Research Branch Agriculture and Agri-Food.
7. Yaalon DH, Yaron B, 1966. Framework for man-made soil changes – an outline of metapedogenesis. *Soil Sci 102:* 272–277.
8. Price EW, Plant DA, 1990. The significance of particle size of soils as a risk factor in the etiology of podoconiosis. *Trans R Soc Trop Med Hyg 84:* 885–886.
9. Deribe K, Brooker SJ, Pullan RL, Hailu A, Enquselassie F, Reithinger R, Newport M, Davey G, 2013. Spatial distribution of podoconiosis in relation to environmental factors in Ethiopia: a historical review. *PLoS ONE 8:* e68330.
10. Bartholomew DJ, Steele F, Moustaki I, Galbraith JI, 2008. Prinicpal components analysis. *Analysis of Multivariate Social Science Data*. Boca Raton, FL: Taylor & Francis Group, LLC.
11. Lee S, Batzoglou S, 2003. Application of independent component analysis to microarrays. *Genome Biol 4:* R76.
12. Jolliffe IT, 1972. Discarding variables in a principal component analysis I: artificial data. *Appl Statist 21:* 160–173.
13. Korkeila EA, Sundström J, Pyrhönen S, Syrjänen K, 2011. Carbonic anhydrase IX, hypoxia-inducible factor-1α, ezrin and glucose transporter-1 as predictors of disease outcome in rectal cancer: multivariate Cox survival models following data reduction by principal component analysis of the clinicopathological predictors. *Anticancer Res 31:* 4529–4535.
14. Price EW, 1974. The relationship between endemic elephantiasis of the lower legs and the local soils and climate. *Trop Geogr Med 26:* 225–230.
15. Price EW, Bailey D, 1984. Environmental factors in the etiology of endemic elephantiasis of the lower legs in tropical Africa. *Trop Geogr Med 36:* 1–5.
16. Sturrock HJ, Gething PW, Clements AC, Brooker S, 2010. Optimal survey designs for targeting chemotherapy against soil-transmitted helminths: effect of spatial heterogeneity and cost-efficiency of sampling. *Am J Trop Med Hyg 82:* 1079–1087.
17. Srividya A, Michael E, Palaniyandi M, Pani SP, Das PK, 2002. A geostatistical analysis of the geographic distribution of lymphatic filariasis prevalence in southern India. *Am J Trop Med Hyg 67:* 480–489.

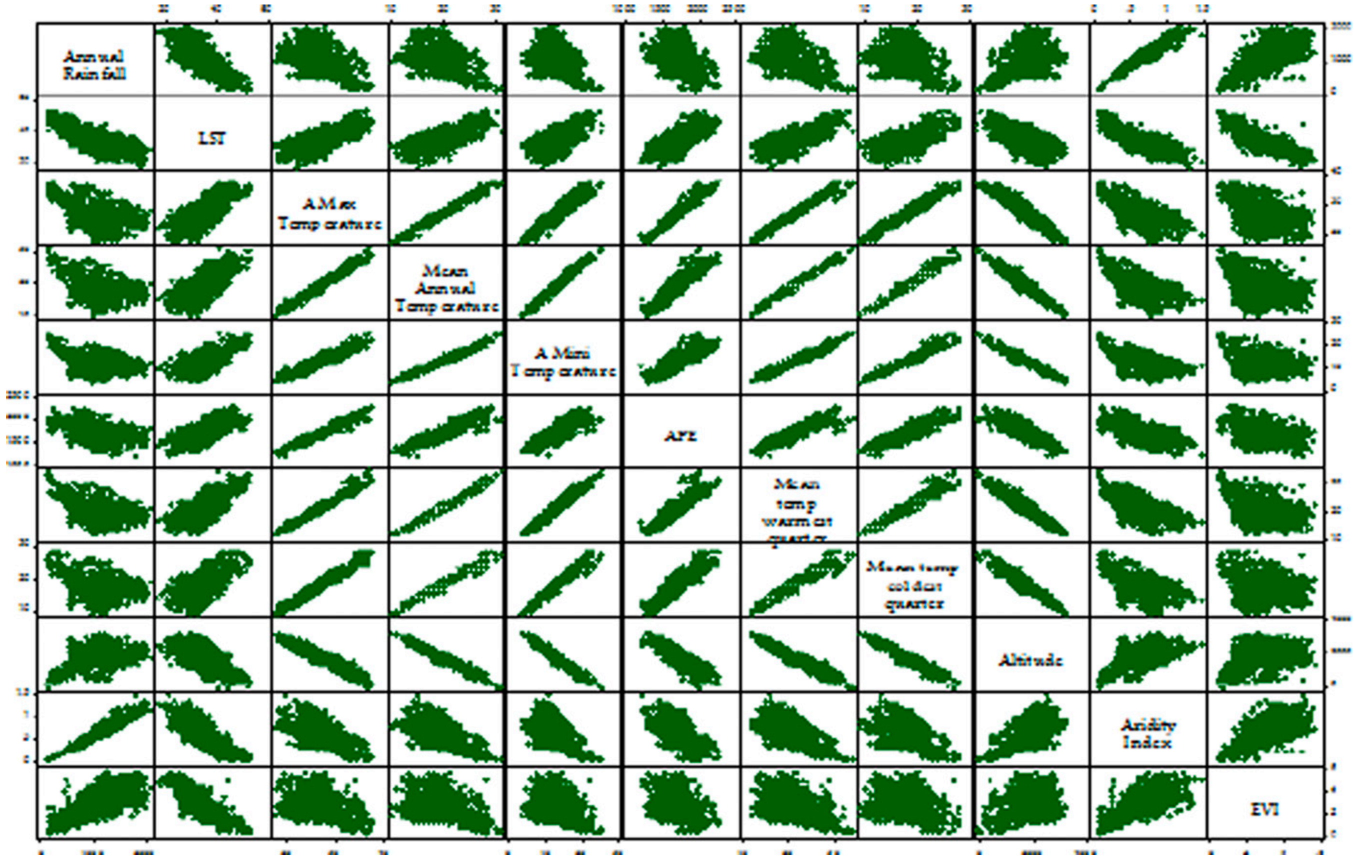

Figure S1. Scatter matrix of climatic and environmental variables showing correlation of variables.

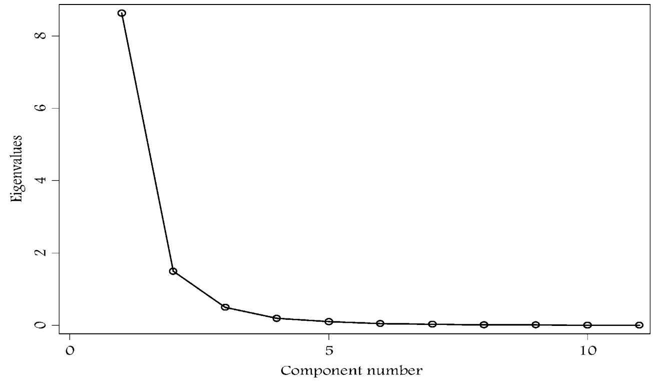

Figure S2. Scree plot of the variances of the principal components. Only the two comports have eigenvalue > 1. The elbow of the plot appears near the third component, suggesting the first two components explain most of the variations.

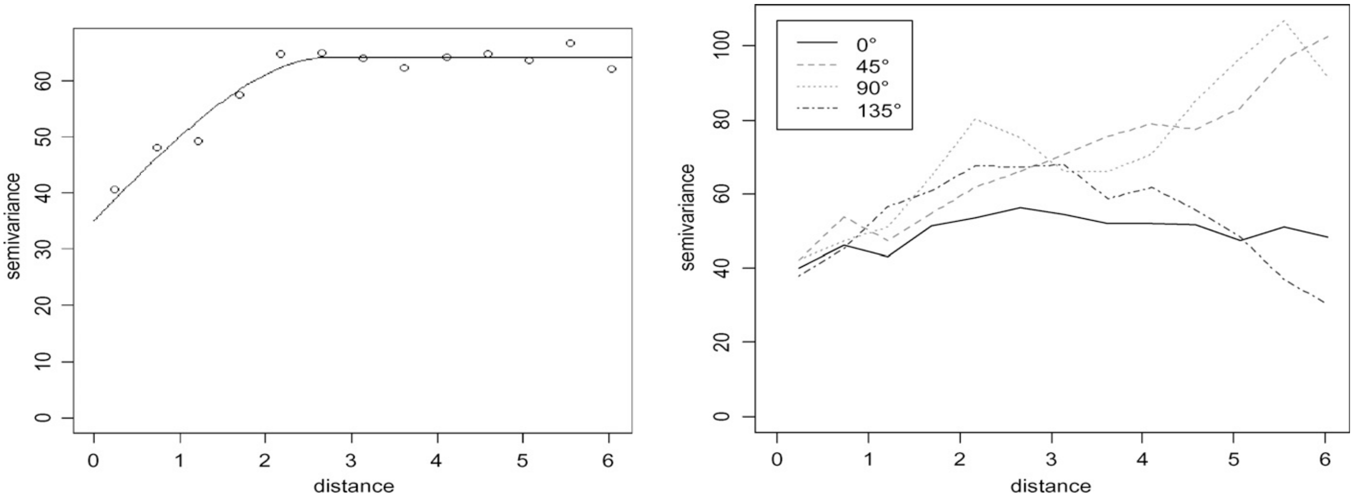

Figure S3. Empirical semivariograms model of the spatial dependency of podoconiosis in Ethiopia and best-fit lines of spherical spatial raw prevalence data. From 1,315 villages, among adults >, =,15 years of age in Ethiopia. Parameter values fitted were range = 2.795998, sill = 58.3286, nugget.

Supplemental Table 1

Correlation matrix of environmental and climate variables

Variable	Mean annual rainfall	LST	Annual maximum temperature	Mean annual temperature	Annual minimum temperature	APE	Mean temperature in the warmest quarter	Mean temperature in the coldest quarter	Altitude	Aridity index	EVI
Mean annual rainfall	1										
LST	–0.7058	1									
Annual maximum temperature	–0.553	0.7813	1								
Mean annual temperature	–0.5545	0.7661	0.9839	1							
Annual minimum temperature	–0.546	0.7371	0.9454	0.9829	1						
APE	–0.5154	0.7469	0.971	0.9191	0.8453	1					
Mean temperature in the warmest quarter	–0.5624	0.7895	0.9792	0.9868	0.9717	0.9143	1				
Mean temperature in the coldest quarter	–0.5132	0.7178	0.9693	0.9807	0.9703	0.9087	0.9556	1			
Altitude	0.5414	–0.7341	–0.9541	–0.9749	–0.9737	–0.8845	–0.9563	–0.9709	1		
Aridity index	0.9687	–0.7751	–0.7006	–0.6843	–0.6537	–0.6818	–0.6905	–0.6458	0.6662	1	
EVI	0.6579	–0.7866	–0.4447	–0.4146	–0.3812	–0.4343	–0.4584	–0.3519	0.3578	0.6576	1

LST = land surface temperature; APE = annual potential evapotranspiration; EVI = enhanced vegetation index.

Supplemental Table 2

Eigenvalues proportions explained by each components and loadings of the principal components*

Variable	Comp1	Comp2	Comp3	Comp4	Comp5	Comp6	Comp7	Comp8	Comp9	Comp10	Comp11
Mean annual rainfall	–0.2409	0.4874	0.5304	0.0914	0.0223	–0.0314	–0.0963	–0.6179	0.1088	0.0829	0.058
LST	0.2955	–0.2625	0.3782	–0.1136	0.8236	–0.0023	0.0976	0.0012	0.0035	–0.0005	0.0036
Annual maximum temperature	0.3311	0.1598	0.0572	0.2562	–0.0615	0.1173	0.0559	0.0272	0.0603	0.219	–0.8503
Mean annual temperature	0.3304	0.1832	0.0051	–0.0995	–0.0904	0.1586	0.0933	0.0275	0.68	–0.5811	0.0674
Annual minimum temperature	0.3222	0.1972	–0.0413	–0.4607	–0.1123	0.1373	0.1148	0.054	0.2261	0.6936	0.252
APE	0.3144	0.1397	0.091	0.7661	0.0012	–0.0605	–0.0677	0.2609	0.0334	0.1658	0.4262
Mean temperature in the warmest quarter	0.3302	0.1493	0.0673	–0.0923	–0.0924	0.6588	–0.155	–0.0973	–0.5604	–0.2261	0.1196
Mean temperature in the coldest quarter	0.3221	0.2312	–0.0362	–0.0759	–0.1099	–0.4923	0.6256	–0.146	–0.3666	–0.1784	0.0481
Altitude	–0.3227	–0.2071	0.0737	0.2256	–0.0381	0.4956	0.7257	–0.0459	0.098	0.09	0.0558
Aridity index	–0.2787	0.385	0.4434	–0.1861	–0.0163	–0.0076	0.0789	0.7159	–0.1082	–0.0884	–0.0646
EVI	–0.1964	0.5561	–0.5954	0.0655	0.5237	0.1245	0.0571	0.0146	–0.0093	–0.0025	–0.01
Eigenvalues	8.63	1.49	0.49	0.19	0.103	0.042	0.026	0.008	0.006	0.003	0.001
Cumulative proportion of variance	0.78	0.92	0.96	0.98	0.99	0.996	0.998	0.999	0.999	0.999	1.000

*As indicated below the first two components explained 92% of the variation and variables were selected from these two components. The highlighted variables were selected from the two components based on previous literature and loading values.

LST = land surface temperature; APE = annual potential evapotranspiration; EVI = enhanced vegetation index.

Supplemental Table 3

Univariate multilevel mixed-effects logistic regression model developed using a likelihood-based approach, with random intercepts for village and woreda for podoconiosis among individuals ≥ 15 years of age in Ethiopia

Variable	Category	Non-cases Number (%)	Podoconiosis cases Number (%)	Univariate
Sex	Male	62650 (96.4)	2208 (3.4)	1.0
	Female	62056 (95.3)	3045 (4.7)	1.5 (1.4–1.6)
Age in years	Mean(SD)	36.1 (15.3)	44.4 (16.6)	1.02 (1.01–1.02)
Education	No formal education	73262 (94.6)	4162 (5.4)	1.0
	Primary 1–8	33444 (97.4)	910 (2.6)	0.4 (0.4–0.5)
	Secondary 9–12	13765 (98.8)	166 (1.2)	0.2 (0.2–0.3)
	Post-secondary > 12	4235 (99.7)	15 (0.3)	0.1 (0.04–0.1)
Marital status	Married	91912 (96.1)	3722 (3.9)	1.0
	Unmarried ¥	32794 (95.5)	1531 (4.5)	1.4 (1.3–1.5)
Religion	Muslim	44074 (98.3)	752 (1.7)	1.0
	Other	80632 (94.7)	4501 (5.3)	1.4 (1.2–1.6)
Type of floor	Mud/earth	115354 (92.5)	5145 (4.3)	1.0
	Cement/wood/plastic	9352 (98.9)	108 (1.1)	0.4 (0.3–0.5)
Wore shoes before age 12?	Yes	76725 (98.4)	1286 (1.6)	1.0
	No	47981 (92.4)	3967 (7.6)	2.9 (2.7–3.1)
How often do you wash your legs?	Daily or more often	86533 (95.9)	3754 (4.1)	1.0
	Two-three times a week	37379 (96.6)	1325 (3.4)	1.1 (1.0–1.2)
	Weekly or less often	794 (82.0)	174 (18.0)	4.2 (3.3–5.3)
Occupation:	Professional	8988 (99.3)	68 (0.7)	1.0
	Semiskilled	77366 (95.7)	3488 (4.3)	3.9 (3.0–5.0)
	Unemployed	38352 (95.8)	1705 (4.2)	4.4 (3.4–5.7)
Variables		Non-cases	Podoconiosis cases	Mean difference
Age in years	Mean (SD)	36.1 (15.3)	44.4 (16.6)	–8.6
Monthly household income	Mean (IQR)	615.9 (200–700)	322.8 (100–430)	N/A
Age at first shoe wearing	Mean (SD)	11.4 (9.2)	21.4 (14.5)	–9.9
Shoe wearing duration in years	Mean (SD)	24.2 (13.7)	22.4 (14.8)	1.8
Environmental Variables	Categories			Univariate
EVI	< 0.2			1.0
	0.2–0.4			2.3 (1.6–3.4)
	> 0.4			5.1 (2.9–9.1)
Rainfall	< 1,000			1.0
	> = 1,000			3.3 (2.5–4.3)
Altitude	< 1,000			1.0
	1,000–2,800			4.5 (2.2–9.5)
	> 2,800			0.4 (0.1–1.1)

EVI = enhanced vegetation index.
¥ = Single, divorced, widowed.
